# Effects of Aspirin on Odontogenesis of Human Dental Pulp Cells and TGF-*β*1 Liberation from Dentin *In Vitro*

**DOI:** 10.1155/2022/3246811

**Published:** 2022-08-05

**Authors:** V. Khampatee, C. Zhang, L. Chou

**Affiliations:** Department of Restorative Sciences and Biomaterials, Boston University Henry M. Goldman School of Dental Medicine, Boston, MA, USA

## Abstract

**Aim:**

This *in vitro* study aimed to investigate the roles of aspirin (ASA) and its concentrations on the odontogenesis of human dental pulp cells (HDPCs) and to investigate the influence of ASA on TGF-*β*1 liberation from dentin. *Methodology*. HDPCs were cultured in a culture medium with 25, 50, 75, 100, and 200 *μ*·g/mL ASA and 0 *μ*·g/mL ASA as a control. The mitochondrial activity of HDPCs was assessed using an MTT assay. Crystal violet staining and triton were used to evaluate cell proliferation rates. ALP activity was measured with a fluorometric assay. Expressions of DSP and RUNX2 were determined with the ELISA. DSP and RUNX2 mRNA levels were measured with RT-qPCR. Alizarin red staining was conducted to evaluate the mineralized nodule formation. Dentin slices were submerged in PBS (negative control), 17% EDTA (positive control), and ASA before collecting the solution for TGF-*β*1 quantification by the ELISA. The data were analyzed by the *t-*tests and ANOVA, followed by the Tukey post hoc tests. *P* values < 0.05 were considered statistically significant.

**Results:**

The results showed that 25–50 *μ*·g/mL ASA promoted mitochondrial activity of HDPCs at 72 h (*P* < 0.05) and yielded significantly higher proliferation rates of HDPCs than the control at 14d and 21d (*P* < 0.001). All concentrations of ASA promoted odontogenic differentiation of HDPCs by enhancing the levels of DSP and RUNX2, their mRNA expression, and mineralization in a dose-dependent manner. Also, ASA yielded significantly higher TGF-*β*1 liberation after conditioning dentin for 5 min (25, 200 *μ*·g/mL; *P* < 0.001) and 10 min (200 *μ*·g/mL; *P* < 0.05).

**Conclusions:**

This *in vitro* study demonstrated that ASA, especially in high concentrations, promoted the odontogenesis of HDPCs and TGF-*β*1 liberation from dentin, showing the potential of being incorporated into the novel pulp capping materials for dental tissue regeneration.

## 1. Introduction

Acetylsalicylic acid (ASA), aspirin, is a renowned nonsteroidal anti-inflammatory drug (NSAID) and antithrombotic agent used for decades to treat inflammatory and cardiovascular conditions [1]. Recently, an association between increased bone mineral density and the use of ASA was reported in an epidemiological study [2, 3]. Also, the low dose of ASA improved osteogenesis of bone marrow mesenchymal stem cells (BMMSCs) in ovariectomized mice by targeting telomerase activity and blocking osteoclastogenesis [4]. Coadministration of ASA and allogeneic rat adipose-derived stromal cells (rASCs) attenuated bone loss and decreased proinflammatory cytokines in serum [5]. In dentistry, ASA-treated stem cells from exfoliated deciduous teeth (SHED) significantly promoted osteogenic differentiation and immunomodulation by upregulating the telomerase reverse transcriptase (TERT)/Wnt/*β*-catenin cascade [6]. Another study demonstrated the positive effects of ASA on the expression of growth factor-associated genes and the osteogenic capacity of periodontal ligament stem cells (PDLSCs), suggesting the roles of ASA in periodontal health [7]. Furthermore, the odontogenic differentiation of stem cells from the apical papilla (SCAPs) was enhanced by 100 *μ*·g/mL ASA [8]. However, the influence of ASA and its concentration range on the odontogenesis of human dental pulp cells (HDPCs) and dentin remains elusive.

HDPCs are a heterogeneous cell population, including postnatal multipotent mesenchymal stem cells (MSCs) and progenitor cells that exhibit the potential in tissue regeneration [9]. Surrounded by dentin, HDPCs reside in the dental pulp and play a crucial role in dental tissue repair when the teeth are subject to injury [10]. Various conditions such as caries, erosion, and dental restorative procedures or materials can cause dentin demineralization leading to the liberation of growth factors sequestrated within the dentin matrix during tooth development [11, 12]. Among the bioactive molecule reservoir, the transforming growth factor *β* (TGF-*β*) family is believed to obtain a variety of cell signaling properties that invoke dentinogenic responses of HDPCs, including cell proliferation, differentiation, and mineralization to initiate the production of reparative dentin [13]. Despite the natural regenerative capacity of the dentin-pulp complex, an amount of tertiary dentin gained from self-healing is hardly sufficient to maintain the pulp vitality without the need for restorations. Hence, constantly searching for the materials that help synergize the healing potential of the pulp is critical to improving clinical outcomes. Calcium hydroxide (Ca(OH)_2_) and tricalcium silicate cement (TSC) have long been considered options to opt for in pulp capping procedures. Regardless of their popularity and ability to release TGF-*β*1 from dentin via collagen degradation [11], only 60–80% success rates were reported after a 3-year follow-up in randomized clinical trials [14, 15], and long-term exposure to highly alkaline conditions created by Ca(OH)_2_ and TSC could cause local necrosis of the pulp tissue [16] and hinder the mechanical properties of dentin [17, 18]. On the other hand, ASA with the reported bone regenerative capacity and acidity might be an additional ingredient that helps neutralize the pH of current pulp capping materials and supports dental tissue regeneration. This project hypothesizes that ASA at an optimal concentration has an inductive effect on HDPCs' odontogenesis as well as TGF-*β*1 liberation from dentin, which could benefit the development of pulp capping materials.

## 2. Materials and Methods

**Figure 1:**
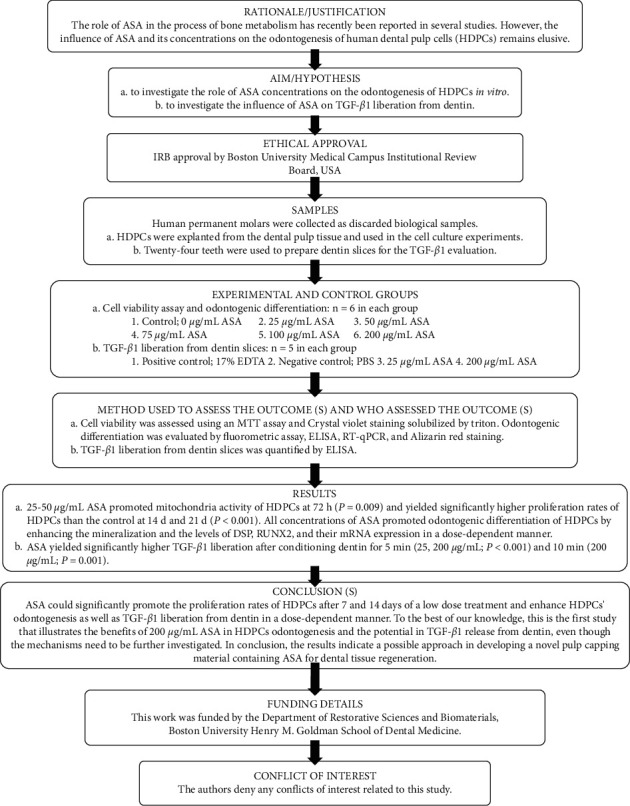
The Preferred Reporting Items for Laboratory studies in Endodontology (PRILE) 2021 flowchart summarizes the key steps in the reporting of a laboratory study.

The manuscript of this laboratory study has been written according to the Preferred Reporting Items for Laboratory studies in Endodontology (PRILE) 2021 guidelines [19] (Figure 1).

### 2.1. Ethical Statement

Human permanent molars used in experiments were considered discarded biological samples and obtained from healthy donors (18–25 years of age) at the Department of Oral and Maxillofacial Surgery, Boston University Henry M. Goldman School of Dental Medicine. The procedures conformed to approved guidelines by the Boston University Medical Campus Institutional Review Board (IRB; H-33173).

### 2.2. Cell Isolation and Culture

HDPCs were isolated from the teeth according to the previous reports [20, 21] with modifications. Briefly, the teeth were cut using sterilized instruments. The pulp tissues were meticulously collected from the teeth and explanted into tissue culture flasks. HDPCs were cultured in Basal Medium Eagle (BME; Thermo Fisher Scientific, Waltham, MA) supplemented with 10% fetal bovine serum (FBS; R&D Systems, Inc., Minneapolis, MN), 100 *U/mL* of premixed antibiotics (Thermo Fisher Scientific), 0.25 *μ*·g/mL amphotericin B (Gibco; Thermo Fisher Scientific), and then incubated at 37°C in 5% CO_2_. The culture medium was changed twice a week, and passage 3 (P3) cells were used for further experiments.

### 2.3. ASA Preparation and pH Evaluation

ASA stock solution was prepared by combining the ASA powder (Sigma-Aldrich, St. Louis, MO) with sterilized phosphate-buffered saline (PBS; Gibco) [8]. The 3-day pH evaluation was conducted to confirm that each concentration of ASA was within the biological range and safe for the HDPCs. The growth medium with ASA concentrations of 25, 50, 75, 100, and 200 *µ*·g/mL was prepared in 15 mL tubes in triplicate with 0 *µ*·g/mL ASA as a control. The pH evaluation was conducted immediately after the preparation using a pH meter with an electrode tip (Thermo Fisher Scientific). The growth medium with all concentrations of ASA was incubated at 37°C in 5% CO_2_, and the pH evaluation was taken at 20 min, day1, day2, and day3 to determine the pH stability of ASA over time.

### 2.4. Attachment Efficiency and Cell Viability Assay

HDPCs were cultured on 24-well plates at a density of 3 × 10^3^ cells/well with culture medium containing 25, 50, 75, 100, or 200 *µ*·g/mL ASA with 0 *µ*·g/mL as a control (*n* = 6). The cell attachment efficiency was determined at 16 h, and the proliferation rate of HDPCs was evaluated after 7, 14, and 21 d of ASA treatment (*n* = 6). HDPCs were fixed with 10% neutral buffered formalin and then incubated with 0.2% crystal violet solution (Sigma-Aldrich). The quantification was performed by solubilizing the crystal violet staining with 1% Triton X-100 solution (Sigma-Aldrich), and the absorbance was measured at 590 nm using a spectrophotometer (Infinite® M1000 Pro Plate Reader; Tecan, Männedorf, Switzerland).

Mitochondrial activity was assessed at 24, 48, and 72 h using the methylthiazolyldiphenyl-tetrazolium bromide (MTT) assay kit (Abcam, Waltham, MA) according to the manufacturer's protocols. HDPCs were cultured on 96-well plates at a density of 7 × 10^3^ cells/well with culture medium containing 25, 50, 75, 100, or 200 *μ*·g/mL ASA with 0 *μ*·g/mL as a control (*n* = 6). At each time point, serum-free medium and 50 *μ*·L of the MTT reagent were added to each well and then incubated at 37°C for 3 h. The reagent was removed, and then, 150 *μ*·L of the MTT solvent was added into each well. The plate was incubated on the shaker at room temperature (RT) for another 15 min, and the absorbance was read at 590 nm.

### 2.5. *In Vitro* Odontogenic Differentiation

HDPCs were cultured on 24-well plates at a density of 3 × 10^3^ cells/well with odontogenic medium containing 0, 25, 50, 75, 100, or 200 *μ*·g/mL ASA. The odontogenic medium consisted of 10% charcoal-stripped FBS (Gibco), 100 U/mL premixed antibiotics (Thermo Fisher Scientific), 10^−8^ M menadione (Sigma-Aldrich), 10 mM *β*-glycerophosphate (Sigma-Aldrich), 0.2 mM L-ascorbic acid (Sigma-Aldrich), and 2 mM L-glutamine (Gibco) in BME (Thermo Fisher Scientific). After 7, 14, and 21 d of induction, the supernatant was harvested to evaluate odontogenic biomarkers, and the cells were stained with alizarin red to examine mineralization.

### 2.6. Odontogenic Biomarkers

Alkaline phosphatase (ALP) activity was detected using the alkaline phosphatase fluorometric assay kit (Abcam) (*n* = 4). According to the manufacturer's protocols, the collected supernatant, standard, and assay buffer as negative control were incubated with a nonfluorescent 4-methylumbelliferyl phosphate disodium salt (MUP) substrate in a 96-well plate for 30 min at RT, protected from light. Stop solution was added to each well, and the fluorescent signal was read at Ex/Em = 360/440 nm.

The human dentin sialoprotein (DSP) and runt-related nuclear factor 2 (RUNX2) enzyme-linked immunosorbent assay (ELISA) kits (Express Biotech International, Frederick, MD) were applied by following the manufacturer's protocols to evaluate the DSP and RUNX2 activities subsequently (*n* = 4). The collected supernatant, standard, and assay buffer as negative control were added to the precoated wells and incubated for 90 min at 37°C. The plate was washed and incubated with a biotin-labeled antibody, an HRP-streptavidin conjugate (SABC), and a TMB substrate accordingly with wash steps in between. The stop solution was added to each well, and the absorbance was read at 450 nm.

### 2.7. Reverse Transcription-Quantitative Polymerase Chain Reaction (RT-qPCR)

HDPCs were treated with odontogenic medium containing ASA for 14 d, and then, the TRIzol reagent (Invitrogen; Thermo Fisher Scientific) was applied to lyse the cells for RNA extraction and purification using PureLink™ RNA Mini Kit incorporating with PureLink™ DNase Set (Invitrogen) according to the manufacturer's instructions. RNA yield and RNA quality were determined by using NanoDrop™ 2000 UV Visible Spectrophotometer (Thermo Fisher Scientific). The extracted RNA of 0.2 *μ*·g from each sample was utilized in one-step RT-qPCR using SuperScript™ III Platinum™ One-Step qRT-PCR Kit (Invitrogen) on StepOnePlus™ Real-Time PCR System (Applied Biosystems; Thermo Fisher Scientific). The target TaqMan probes of dentin sialophosphoprotein (DSPP; Hs00171962_m1; Thermo Fisher Scientific) and RUNX2 (Hs00231692_m1; Thermo Fisher Scientific) were applied. The housekeeping gene, *β*-actin (Hs03023943_g1; Thermo Fisher Scientific), was used to normalize gene expression levels of each target gene, and the 2^−^^ΔΔCt^ calculation was conducted for the relative differences in the gene expressions (*n* = 6). PCR was set under standard cycling conditions as described: 50°C for 15 min followed by 95°C for 2 min, then 40 cycles of 95°C for 15 s, and 60°C for 30 s.

### 2.8. Mineralization

After HDPCs were fixed with 10% neutral buffered formalin, the cells were incubated for 45 min at RT with 500 *μ*·L of 2% alizarin red S (Sigma-Aldrich) adjusted pH to 4.1–4.3 using NH_4_OH. The alizarin red staining was solubilized by incubating with 1.2 mL of 10% acetic acid for 1 h to quantify the mineralized nodule formation, and the absorbance was measured (*n* = 6).

### 2.9. Evaluation of Liberated TGF-*β*1 from Dentin Slices

Dentin slices were prepared according to the previously reported protocol [12]. Human permanent molars were sectioned into the thickness of 0.5–0.6 mm using a linear precision saw (IsoMet® 5000; Buehler, Lake Bluff, IL), and then pulp tissues, cementum, and enamel were removed. The dentin was further cut into slices with an estimated surface area of 1.5 cm^2^ before conditioning with 500 *μ*·L of PBS (negative control), 17% ethylenediaminetetraacetic acid solution (EDTA; Vista Apex, Racine, WI; positive control), or ASA at a concentration of 25 or 200 *μ*·g/mL. After 5 min or 10 min, the solution was collected and stored in a −20°C freezer for further TGF-*β*1 evaluation using the human TGF-*β*1 ELISA kit (Thermo Fisher Scientific) according to the manufacturer's protocols (*n* = 5).

### 2.10. Statistical Analysis

Statistical analyses were performed using JMP Pro version 15.2 software (SAS Institute Inc., Cary, NC). Comparisons between two groups were analyzed by independent 2-tailed Student's *t*-tests, and multiple groups comparisons were acquired by one-way analysis of variance (ANOVA) followed by the Tukey post hoc tests. *P* values < 0.05 were considered statistically significant.

## 3. Results

### 3.1. ASA pH Evaluation

**Figure 2:**
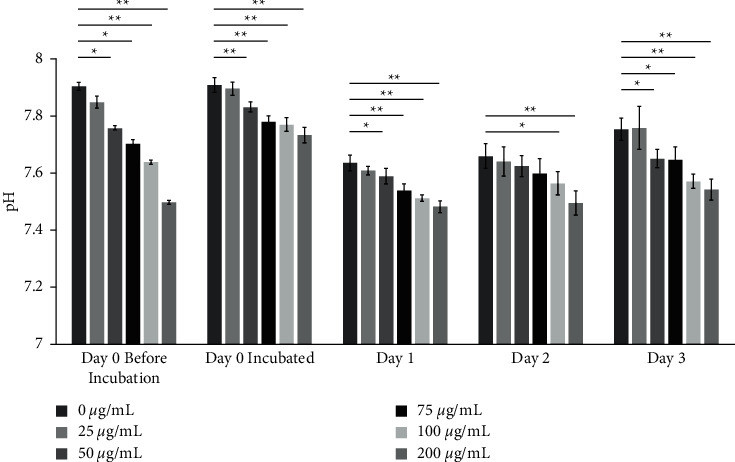
Acetylsalicylic acid (ASA) 3-day pH evaluation showed that the pH of the ASA-treated culture medium was within the biological range for the human dental pulp cells (HDPCs) culture. *n* = 3 in each group. ^*∗*^*P* < 0.05. ^*∗∗*^*P* < 0.001. Values are presented as mean ± SD.

The highest concentration of ASA for the experiments, 200 *μ*·g/mL, was determined based on the minimum plasma concentration that early signs of salicylic overdose occurred, according to the United States Food and Drug Administration (FDA), Code of Federal Regulations (CFR) Title 21 CFR 343. After preparing ASA, a 3-day pH evaluation was conducted to ensure that the pH of all ASA concentrations in the culture medium was within the biological range to conduct the cell cultures (Figure 2). ASA concentrations over 25 *μ*·g/mL significantly lowered the pH of the culture medium from the control in a dose-dependent manner (*P* < 0.006). The incubator condition kept the pH stable at a range of 7.5–7.7 throughout the evaluation.

### 3.2. Effects of ASA on HDPCs' Attachment Efficiency and Viability

**Figure 3:**
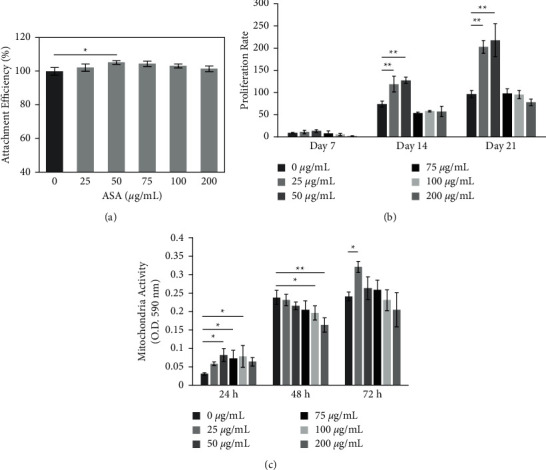
Acetylsalicylic acid (ASA) treatment showed no cytotoxic effect on human dental pulp cells (HDPCs). (a, b) Crystal violet staining solubilized by triton X-100 showed significantly higher attachment efficiency of HDPCs-treated with 50 mg/mL ASA than the control at 16h. A significant increase in the proliferation rate was observed in HDPCs treated with low concentrations of ASA (25–50 mg/mL). (c) MTT assay indicated that ASA-treated HDPCs expressed an elevation of mitochondria activity over time, and HDPCs treated with 25 mg/mL ASA showed a significantly higher mitochondria activity at 72h compared to the control. *n* = 6 in each group. ^*∗*^*P* < 0.05. ^*∗∗*^*P* < 0.001. Values are presented as mean ± SD.

HDPCs treated with each concentration of ASA reached the highest attachment level at 16 h, and 50 *μ*·g/mL ASA exhibited significantly higher attachment efficiency than the control (*P* = 0.027; Figure 3(a)). The mitochondrial activity observed with the MTT assay showed that all concentrations of ASA were nontoxic to HDPCs as the cell numbers increased within the experimental period (Figure 3(c)). At 72 h, the mitochondrial activity was significantly enhanced by using 25 *μ*·g/mL ASA (*P* = 0.009), which was also consistent with our 3d proliferation (data not shown). Longer observation by crystal violet staining solubilized by Triton X-100 demonstrated that 25–50 *μ*·g/mL ASA yielded significantly higher proliferation of HDPCs than the control at 14 and 21 d (*P* < 0.001), with 50 *μ*·g/mL ASA representing the peak proliferation, whereas concentrations of ASA over 75 *μ*·g/mL downregulated the proliferation of HDPCs, with the lowest point at 200 *μ*·g/mL ASA (Figure 3(b)). The proliferation rate was calculated based on the cell attachment efficiency of each group at 16 h. The HDPCs in all groups showed a spindle-like shape, and no significant morphology changes in the ASA-treated groups were detected over time.

### 3.3. ASA Promotes HDPCs' Odontogenic Differentiation *In Vitro*

**Figure 4:**
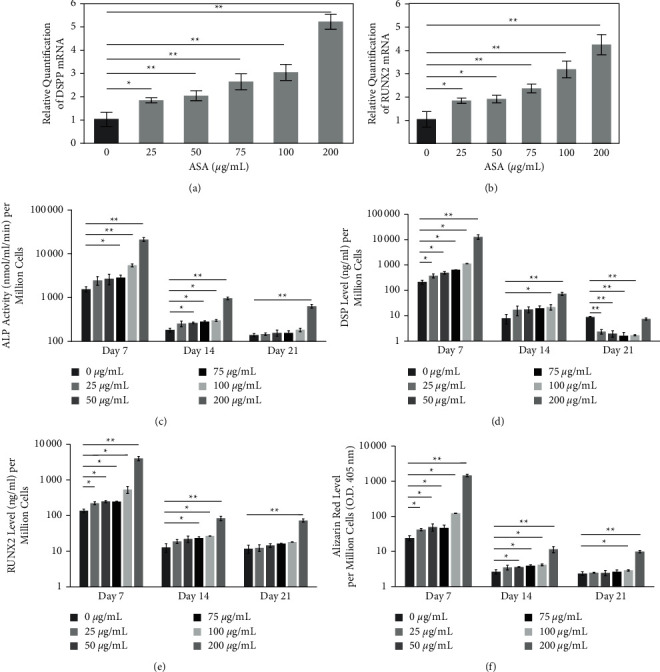
The effects of acetylsalicylic acid (ASA) treatment enhanced the odontogenic capacity of human dental pulp cells (HDPCs) in a dose-dependent manner. (a, b) Reverse transcription-quantitative polymerase chain reaction (RT-qPCR) demonstrated the dentin sialophosphoprotein (DSPP) and runt-related nuclear factor 2 (RUNX2) gene expression levels of HDPCs at 14 d of odontogenic induction (*n* = 6). Results showed the ratios to the control. (c-e) Alkaline phosphatase (ALP), dentin sialoprotein (DSP), and RUNX2 biomarker levels were evaluated after 7, 14, and 21 d of odontogenic induction (*n* = 4). (f) Alizarin red staining showed that high concentrations of ASA significantly promoted the mineralization of HDPCs after 7, 14, and 21 d of treatment (*n* = 6). The biomarker and alizarin red levels were normalized to cell numbers per million cells showing that the production per cell decreased over time. ^*∗*^*P* < 0.05. ^*∗∗*^*P* < 0.001. Values are presented as mean ± SD.

RT-qPCR was used to detect the odontogenic capacity of HDPCs at the transcriptional level. After normalization with *β*-actin, the gene expression of HDPCs treated with concentrations of ASA for 14 d showed a significant elevation of DSPP and RUNX2 genes in a dose-dependent manner compared to the control (Figures 4(a)-4(b)), which supported the biomarker results.

The levels of ALP, DSP, and RUNX2 in the supernatant were assessed to elucidate HDPCs' odontogenic differentiation at the post-transcriptional level. At each time point, the result showed that all concentrations of ASA upregulated the ALP, DSP, and RUNX2 levels per cell in a dose-dependent manner (Figures 4(c)–4(e)), with 200 *μ*·g/mL ASA markedly enhanced all biomarkers (*P* < 0.001), which conformed to gene expression levels and the mineralized nodule deposition (Figure 4(f)). The DSP level at 21 d did not follow the same trend. However, the overall production of all biomarkers and calcium deposition per cell declined from markedly distinct elevation at 7 d to the lower level at 21 d by several folds.

### 3.4. ASA Enhances TGF-*β*1 Liberation from ASA-Treated Dentin Slices

**Figure 5:**
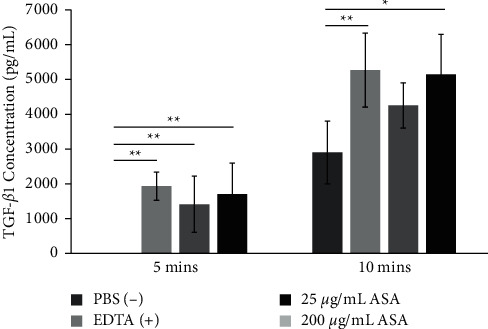
Transforming growth factor *β*1 (TGF-*β*1) concentration in the supernatant following 5min and 10min of dentin slice conditioning with phosphate-buffered saline (PBS; negative control), ethylenediaminetetraacetic acid (EDTA; positive control), or concentrations of acetylsalicylic acid (ASA) showed that ASA enhanced TGF-*β*1 release from dentin in a dose-dependent manner. *n* = 5 in each group. ^*∗*^*P* < 0.05. ^*∗∗*^*P* < 0.001. Values are presented as mean ± SD.

The data suggested that ASA had the potential to release TGF-*β*1 from dentin with ASA enhanced the TGF-*β*1 liberation in a dose-dependent manner, and the levels increased over time (Figure 5). ASA 200 *μ*·g/mL demonstrated a comparable potential to the positive control (EDTA) after 10 min-conditioning. At 5 min, there was no TGF-*β*1 detected in the supernatant after PBS conditioning. The TGF-*β*1 concentration increased at 10 min-PBS conditioning, although the level was significantly lower than the level released by EDTA (*P* < 0.001) and ASA 200 *μ*·g/mL (*P* = 0.001).

## 4. Discussion

ASA has been reported to show positive outcomes in osteogenic induction in cell therapy and bone tissue engineering [22–24]. We speculate that ASA, with its chemical and therapeutic properties, could potentially enhance the odontogenesis of HDPCs and might be another promising element to develop novel pulp capping materials.

HDPCs compose of a heterogeneous population, including postnatal MSCs confined within the pulp chamber [9]. When the teeth are subject to an injury, HDPCs play a crucial role in sustaining the damage by generating the tertiary dentin [10, 13]. In this study, the HDPCs were obtained without a preselection of dental pulp stem cells (DPSCs) or a picked colony culturing method for lineage-specific differentiation. It is because the purpose of the study is to evaluate the potential of ASA to be utilized in the future development of materials for a clinical setting such as a dental pulp capping procedure and that the chance for the material to be directly exposed to HDPCs in the pulp tissue is high. Hence, the use of heterogeneous HDPCs would be suitable to provide more clinically relevant results. Also, recent studies have demonstrated the multilineage differentiation potential of HDPCs, and no significant difference was found between multicolony derived and picked colony culture regarding stem cell capacity and cell expansion [21, 24, 25].

To determine the therapeutic potential and the possibility of ASA utilization in dental tissue regeneration, investigation of biocompatibility and the influence of ASA on HDPCs are crucial. The maximum concentration of ASA in this study was 200 *μ*·g/mL, which was declared by the FDA to be the minimal plasma concentration that early salicylism could occur, and other concentrations were within the *in vivo* therapeutic range [1, 26]. Previous studies have reported that a proper pH-conditioned medium for human cell cultures ranges from 7.0–7.8, with the most optimal pH 7.2 for human osteoblasts and pH 7.8 for significantly enhanced cell activities in HDPCs [27, 28]. In this study, the end concentrations of ASA in the culture medium manifested pH of 7.5–7.7, which was within the optimal range for HDPCs and might be another explanation for the elevated ALP levels and mineralization results in HDPCs according to the previous study [28].

In terms of cell viability, preceding evidence indicated that concentrations of NSAIDs largely influenced their effects on MSCs [26, 29]. PDLSCs treated with different concentrations of ASA showed declined proliferation rates in a dose-dependent manner over time [7]. Low concentrations of ASA (<100 *μ*·g/mL) demonstrated no cytotoxic effects on BMSCs, while higher concentrations at 150 *μ*·g/mL showed an inhibitory effect on BMSC proliferation [22]. This report is in line with the present study that, within the concentration range, ASA was nontoxic to HDPCs but may manifest a downregulating effect in high concentrations. Interestingly, another contradicted report showed decreased DPSC viability by an ASA dose over 100 *μ*·g/mL [24].

The significance found in this study could support the hypothesis that ASA could enhance the odontogenesis of HDPCs suggesting the potential of ASA in dental pulp regeneration. The study proved that ASA, especially in high concentration (200 *μ*·g/mL), markedly promoted the gene expression levels of RUNX2 and DSPP in HDPCs. The RUNX2 and DSPP are known to play crucial roles in odontogenic differentiation and dentin formation [30, 31]. The results were consistent with the mineralization and the odontogenic biomarkers of ALP, DSP, and RUNX2, especially at 7 d and 14 d, in which the levels were increased by using ASA in a dose-dependent manner. Considering that the proliferation rate of HDPCs declined as the ASA concentration rose, we speculate that high concentrations of ASA caused the cells to ease down the proliferation and proceed into the differentiation. The same phenomenon has been reported in the ASA-induced osteogenic differentiation in DPSCs [24]. Also, the study found that the peak biomarker and mineralization levels per cell of all groups were found at 7 d and substantially declined over time, which might suggest an early differentiation of HDPCs. The phenomenon was supported by a previous study that reported a pattern of an odontogenic induction of 100 *μ*·g/mL ASA-treated SCAPs [8]. Nevertheless, the present study firstly demonstrated that 200 *μ*·g/mL ASA had the potential to enhance HDPC's odontogenesis, which is opposed to some previous findings that found ASA over 100 *μ*·g/mL cytotoxic [6, 22, 24].

Supporting evidence on the association of ASA and osteogenic differentiation has been reported on other cell types. A low dose of ASA significantly enhanced telomerase activity in the TERT/Wnt/*β*-catenin cascade, leading to an improved stem cell function and bone formation [6, 34]. The treatment of ASA showed the immunomodulatory capacity by reducing interferon-*γ* (IFN-*γ*) and the tumor necrosis factor-*α* (TNF-*α*) in MSC-based tissue regeneration [32, 33]. In addition, an elevated expression of growth factor-associated genes, including FGFs and BMPs, was found in PDLSCs after treatment with 180 *μ*·g/mL of ASA for 24 h [7]. Another study also showed the relation between ASA treatment and the activation of the phosphoinositide 3-kinase-AKT signaling pathway (PI3K-AKT), leading to the odontogenic differentiation of SCAPs [8]. It is suspected that these mechanisms could be applied to ASA-treated HDPCs, but further investigations and *in vivo* studies need to be conducted.

This study is the first discovery that ASA significantly enhanced TGF-*β*1 liberation from dentin, which supports the second hypothesis of this study. The property could be beneficial for vital pulp treatments as the TGF-*β*1 was found primarily in tertiary dentin, suggesting their crucial roles in dental tissue response to injury [34]. During the restorative procedure, the dentinal tubules serve as channels between the indirect pulp capping material and pulp. Once sequestrated TGF-*β*1 is released from dentin, it may commute through the dentinal tubules into the pulp and promote odontoblast-like cell differentiation from progenitor cells for dentin repair [35, 36]. The TGF-*β*1 extraction from the dentin powder is the property found in Ca(OH)_2_ and TSC. However, TGF-*β*1 extracted from dentin slice condition is minimal [11, 12]. An attempt to liberate the bioactive molecules from dentin slices using dental etchants/conditioners was reported, but the solutions were unable to extract TGF-*β*1 [12]. In this study, 200 *μ*·g/mL ASA could successfully release TGF-*β*1 into an aqueous environment, and the TGF-*β*1 concentrations increased over time to the comparable amount as the positive control at 10 min. Although further investigation on the mechanism is required, this is an interesting finding since the previous study reported the denaturation of bioactive molecules in a prolonged treatment time [12]. It is acknowledged that the conditioning times used in this study may not be feasible in the clinical setting. However, the finding might provide fundamentals for the potential application of ASA in the pulp capping materials and protocols.

## 5. Conclusion

The present study demonstrates that ASA could significantly promote the proliferation rates of HDPCs after 7 d and 14 d of a low-dose treatment (25–50 *μ*·g/mL ASA) and enhance HDPCs' odontogenesis as well as TGF-*β*1 liberation from dentin in a dose-dependent manner. To the best of our knowledge, this is the first study that illustrates the benefits of 200 *μ·*g/mL ASA in HDPCs' odontogenesis and the potential in TGF-*β*1 release from dentin, even though the mechanisms need to be further investigated. In conclusion, the results indicate a possible approach in developing a novel pulp capping material containing ASA for dental tissue regeneration.

## Data Availability

The data that support the findings of this study are available from the corresponding author upon reasonable request.
